# Development of a rating scale for measuring resistance to persuasive health messages

**DOI:** 10.1265/ehpm.22-00059

**Published:** 2022-05-21

**Authors:** Machi Suka, Takashi Shimazaki, Takashi Yamauchi, Hiroyuki Yanagisawa

**Affiliations:** 1 Department of Public Health and Environmental Medicine, The Jikei University School of Medicine, 3-25-8 Nishi-Shimbashi, Minato-ku, Tokyo 105-8461, Japan; 2 The Jikei University School of Medicine, 3-25-8 Nishi-Shimbashi, Minato-ku, Tokyo 105-8461, Japan

**Keywords:** Health message, Rating scale, Disengagement, Reactance, Reliability, Validity, Japan

## Abstract

**Background:**

Pretesting is the key to understanding how the intended audience will react to the message. Resistant reactions affect message processing or can lead to undesirable boomerang effects. The objective of this study was to develop a rating scale for measuring active (reactance) and passive (disengagement) resistance to persuasive health messages.

**Methods:**

Six candidate items (3 items for disengagement and 3 items for reactance) were generated based on literature review. A web-based survey was conducted among Japanese adults aged 25–64 years to verify the reliability and validity of the 6-item resistance scale. Participants were asked to rate one of the advance care planning (ACP) promotion messages. All scale items were scored on a 1-to-5 point Likert scale and they were averaged to produce the resistance score.

**Results:**

Explanatory factor analysis revealed a two-factor solution that agreed with the disengagement and reactance domains, respectively. Correlation coefficients between each set of items ranged between 0.30–0.69. Cronbach alpha (0.86) indicated satisfactory internal consistency of the set of items. Confirmatory factor analysis showed a good fit of the two-factor model with CFI = 0.998, SRMR = 0.011, and RMSEA = 0.041. The resistance score showed a moderate positive correlation with negative emotional responses (displeasure γ = 0.55, anger γ = 0.53) and was significantly inversely related to the persuasiveness score (γ = −0.50). Multivariable logistic regression analysis showed that the odds ratio for ACP intention per 1-point increase in the resistance score was 0.47 (95% confidence interval 0.40–0.56) with adjustment for the persuasiveness score.

**Conclusion:**

The 6-item resistance scale exhibited adequate reliability and validity for measuring audience resistance when applied to the ACP promotion messages in Japanese people. The scale will be useful for pretesting health messages to make them more acceptable to the intended audience.

**Trial registration:**

Not applicable; this is not a report of intervention trial.

**Supplementary information:**

The online version contains supplementary material available at https://doi.org/10.1265/ehpm.22-00059.

## Backgrounds

The novel coronavirus, COVID-19, has raised serious concerns worldwide. Since the virus is primarily transmitted between people through respiratory droplets and contact routes, public health authorities send out messages repeatedly to persuade the general public to engage in infection preventive actions such as physical distancing, personal hygiene, face masks, and vaccination [1]. However, a substantial part of the population have poor adherence to the recommended preventive behaviors [2–4]. In order to tackle this problem, health communication researchers have investigated why many people fail to comply with persuasive health messages. They reveal the existence of two types of psychological barriers, that is, reactance [5–7] and apathy [8, 9] toward persuasive health communication. These resistant reactions will affect message processing and hinder optimal decision making, and in some cases can lead to undesirable boomerang effects.

Brehm’s psychological reactance theory is a commonly utilized framework for understanding resistant reactions to persuasive health messages [10, 11]. People living in liberal cultures value autonomy, or freedom of choice. When a persuasive health message is perceived to threaten one’s freedom of choice, psychological reactance is hypothesized to occur. Recent studies provide a new perspective on audience resistance in the context of message processing [12–15]. Audience may react to threatened freedom in two distinctive ways that represent two different types of resistance: (1) reactance, or active resistance (i.e. arguing against the message) and (2) disengagement, or passive resistance (i.e., withdrawing attention to the message). In the experiments on anti-obesity messages [12], anti-binge drinking messages [13], anti-tobacco messages [14], and sexual violence bystander intervention messages [15], reactance and disengagement were independently and negatively associated with persuasive outcomes. Not only reactance but also disengagement should be considered when predicting the effect of persuasive health messages.

Since persuasive health communication can fail or succeed, the design of persuasive health messages is of great concern to health communication researchers and practitioners. When the receiver understands the message in the way the sender intended, effective communication occurs. However, individuals interpret a message differently based on their prior experience, knowledge, and values. There is a discrepancy between the sender’s intent and the receiver’s understanding in most cases. Therefore, pretesting is a crucial step in developing effective health messages [16]. It helps ensure that what is designed is really suitable for the intended audience.

A variety of methods have been proposed to measure perceived message effectiveness [17]. Meanwhile, many researchers have attempted to measure psychological reactance to persuasive health messages [10, 11]. There are three commonly used measures of state reactance in the literature: the Dillard and Shen measure [18], the Reactance to Health Warnings Scale [19], and the Salzburger State Reactance Scale [20]. However, to our knowledge, no valid instrument exists to measure disengagement or to measure both disengagement and reactance to persuasive health messages simultaneously.

The objective of this study was to develop a rating scale for measuring active (reactance) and passive (disengagement) resistance to persuasive health messages. A web-based survey was conducted to verify the reliability and validity of the proposed resistant scale when applied to advance care planning (ACP) promotion messages in Japanese people. ACP, or *Jinseikaigi* in Japanese, is a process that supports adults at any age or stage of health in understanding and sharing their personal values, life goals, and preferences regarding future medical care [21]. Since 2018, the Ministry of Health, Labour, and Welfare has launched a public awareness campaign to inform the importance of ACP and encourage people to have ACP conversations [22]. In addition to our previously developed perceived message effectiveness scale [23], the proposed resistance scale will be a useful instrument for pretesting persuasive health messages.

## Methods

### Scale development

In order to develop a measure of audience resistance to persuasive health messages, we conducted literature surveys using international (PubMed) and domestic (Cinii) literature databases. Based on our review of the literature on resistance to persuasion, (1) reactance, or active resistance and (2) disengagement, or passive resistance were identified as major psychological barriers affecting persuasiveness of messages [5–9]. Therefore, we designed a scale consisting of two domains, namely, disengagement and reactance domains. The following 6 items associated with disengagement and reactance were derived from previous studies in Japan [24–28]: 1) apathy, 2) irrelevance, and 3) independence composed the disengagement domain; 4) intrusiveness, 5) antipathy, and 6) protest composed the reactance domain. The questionnaire written in Japanese is shown in Appendix. The response options were from 1 (strongly disagree) to 5 (strongly agree). All item scores (range 1–5 points) were averaged to produce an overall score (i.e. resistance score). The disengagement and reactance domain scores were calculated from their component items in the same manner.

### Reliability and validity assessment

In order to verify the reliability and validity of the proposed resistance scale, a web-based survey was conducted in November 2021 among 3,000 Japanese adults aged 25–64 years. Participants were randomly assigned one of the ACP promotion messages (i.e. visual messages presented in poster form for encouraging people to have ACP conversations) and were asked to evaluate it on the resistance scale. The study protocol was approved by the ethics committees of the Graduate School of Information and Communication and has been conducted in accordance with the Ethical Guidelines for Medical and Biological Research Involving Human Subjects by the Japanese Government.

#### Participants

Participants in the survey were recruited from an online research panel of a leading research company in Japan (Rakuten Insight Inc., Tokyo, Japan). Recruitment emails were sent to randomly selected eligible registrants. Since the ACP promotion messages were used as a test object in this study, the following people were excluded through a prescreening process: 1) medical professionals, 2) those who have experienced a life-threatening illness, and 3) those who have heard of ACP, or *Jinseikaigi* in Japanese. Applicants for participation in the survey were accepted in the order of receipt until the number of participants reached the quotas for gender and 10-year age groups (375 people per group). All participants voluntarily agreed to participate in the survey after reading a description of the purpose and procedure of the survey. Consent to participate was implied by the completion and submission of the survey.

Of the 3,000 respondents, 803 people reported that they had seen the ACP poster produced by the Ministry of Health, Labour and Welfare in November 2019. Since previous contact with the ACP poster may have some influence on their responses, the remaining 2,197 respondents (73.2%) were included in this study. Table 1 shows the characteristics of the study participants. The percentages of participants who were married (59.9%) and were employed (79.4%) were almost equal to that of the Japanese population aged 25–64 years (66.0% and 73.0%, respectively in 2015), whereas the percentage of participants with university degrees (48.5%) was considerably higher than that of the Japanese population (21.7% in 2010) [29].

**Table 1:** Characteristics of the study participants

		**N**	
Age	Mean (SD)	45.0	(11.3)
Gender	Men	1144	52.1%
	Women	1053	47.9%
Education	Compulsory education	49	2.2%
	High school	600	27.3%
	Junior college/vocational school	483	22.0%
	University of higher	1065	48.5%
Marital status	Married	1315	59.9%
	Unmarried	699	31.8%
	Divorced/widowed	183	8.3%
Occupation	Full-time job	1374	62.5%
	Temporary or part-time job	371	16.9%
	No occupation	452	20.6%
Household income (million yen †)	<2.00	316	14.4%
	2.00–5.99	980	44.6%
	6.00+	901	41.0%

†1 million yen was about 8,750 U.S. dollars at the time of the survey.

#### Test object

The ACP poster produced by the Ministry of Health, Labour and Welfare in November 2019 was selected as a test object, because there were many arguments for and against the poster on mass media and social media in those days [30, 31]. In addition to the original, two modified versions with different visual designs were created. The original depicts a male comedian lying in hospital bed and regretting not having ACP conversations before. The modified versions contain 1) a photograph of a man riding a motorcycle with a brief description of his ACP experience and 2) only a text message promoting ACP written in black characters on a white background, respectively. The three ACP promotion messages (the original and the modified versions) were used for testing to elicit more diverse responses. Participants in the surveys were randomly assigned one of the three messages (1000 people per message). Participants were asked to see a given message for at least 15 seconds and answer the questions about it. The responses to the three messages were lumped together in this study.

#### Measures

After seeing a given message, participants were asked to what extent the message made them feel 1) surprised, 2) pleasant, 3) angry, 4) fearful, 5) happy, 6) sad, 7) guilty, 8) anxious, and 9) unpleasant [32]. Then participants filled in the persuasiveness scale and the resistance scale. The persuasiveness scale consisting of 7 items has been proven to be reliable and valid in Japanese people [23]. The persuasiveness score was calculated as the average of the items scored on a 1-to-5 point Likert scale; higher scores indicate more positive perception of the message. Moreover, message attention was measured by ‘When the message is hanging on a wall at a station yard, what would you do?’ with 4 response options (not become aware/take no notice/throw a glance/stop to look). ACP intention was measured by asking participants ‘Do you intend having ACP conversations as presented in the message?’ with 4 response options (I have already done/I will do within a month/I will do within six months/I will never do). For analysis, the responses to the ACP intention question were dichotomized into “have no intention (I will never do)” and “will do (I have already done/I will do within a month/I will do within six months)”.

### Statistical analysis

All statistical analyses except for the confirmatory factor analysis were performed using the SAS ver. 9.4 (SAS Institute, Cary, NC, USA). Confirmatory factor analysis was performed using the IBM SPSS Amos ver. 20.0 (IBM Corp, Armonk, NY). Significant levels were set at p < 0.05.

#### Reliability

Explanatory factor analysis with promax rotation was performed to determine the factor structure of the scale. Factor loadings of ≥0.4 were considered to be appropriate. Internal consistency was assessed by Cronbach alpha, where a value of ≥0.7 was considered satisfactory [33].

#### Validity

Confirmatory factor analysis was performed to assess the construct validity of the scale. The strength of relationships between variables was estimated as a standardized regression coefficient or a correlation coefficient. Model fitness was assessed by comparative fit index (CFI), standardized root mean square residual (SRMR), and root mean square error of approximation (RMSEA). For CFI a value closer to 1 indicates better fit (>0.95 is generally considered good), and for SRMR and RMSEA a value closer to 0 indicates better fit (<0.05 is generally considered good) [34].

Criterion validity was evaluated based on the correlation to emotional responses and the inverse relationship to perceived persuasiveness. Pearson correlation coefficient was calculated between the resistance score (and each domain score) and the persuasiveness score. One-way analysis of variance or *t* test was used to compare the mean scores. Construct validity was evaluated by the associations with ACP intention. Since the persuasiveness score can be a predictor of behavioral intention [23], the association between the resistance score and ACP intention should be determined independently of the persuasiveness score. Multivariable logistic regression analysis was performed to calculate odds ratios (ORs) with 95% confidence intervals (CIs) for ACP intention per 1-point increase in the resistance score (and each domain score) with adjustment for the persuasiveness score.

## Results

### Reliability

Table 2 shows the factor structure of the resistance scale. Correlation coefficients between each set of items ranged between 0.30–0.69. The initial factor solution indicated two factors with eigenvalues of 3.15 and 0.59, respectively, which jointly accounted for 100% of the total variance. The promax rotation indicated that the 3 items of the disengagement domain loaded on the first factor and the 3 items of the reactance domain loaded on the second factor.

**Table 2:** Factor structure of the resistance scale

	**Score**		**Factor loadings**		**Communality**
	**Mean**	**SD**	**Factor 1**	**Factor 2**	
Disengagement domain					
Q1) apathy: I am not interested in the topic	3.0	1.0	0.80	−0.06	0.58
Q2) independence: I do not want other people to tell me that	3.1	1.0	0.69	0.07	0.54
Q4) irrelevance: That is irrelevant to me	2.7	0.9	0.68	0.13	0.58
Reactance domain					
Q5) intrusiveness: The message seems intrusive	2.7	1.0	0.32	0.55	0.61
Q3) antipathy: I feel resistance or antipathy	2.5	1.0	0.18	0.73	0.72
Q6) protest: I want to protest against the message sender	2.2	1.0	−0.13	0.91	0.70

The scale items were rated on a scale of 1 (strongly disagree) to 5 (strongly agree).

Table 3 shows the internal consistency of the resistance scale. The percentages of participants with the highest score (0.7%) and the lowest score (2.7%) indicated no ceiling or floor effects. Cronbach alpha and corrected item-total correlation indicated satisfactory internal consistency of the set of items. There was a moderate positive correlation between the two domains scores (γ = 0.59, p < 0.001).

**Table 3:** Internal consistency of the resistance scale

	**Overall/domain ** **score**		**Internal consistency ** **among all items**		**Internal consistency ** **among each domain items**	
	**Mean**	**SD**	**Cronbach ** **alpha**	**Item-total correlation**	**Cronbach ** **alpha**	**Item-total correlation**
Overall	2.69	0.74	0.86			
Disengagement domain	2.92	0.81			0.79	
Q1) apathy: I am not interested in the topic				0.59		0.65
Q2) independence: I do not want other people to tell me that				0.62		0.61
Q4) irrelevance: That is irrelevant to me				0.67		0.64
Reactance domain	2.46	0.85			0.84	
Q5) intrusiveness: The message seems intrusive				0.72		0.67
Q3) antipathy: I feel resistance or antipathy				0.72		0.74
Q6) protest: I want to protest against the message sender				0.58		0.70

Overall/domain scores were calculated as the average of the scale items scored on a 1-to-5 point Likert scale.

Internal consistency was assessed among all items and also among each domain items.

### Validity

**Fig. 1:**
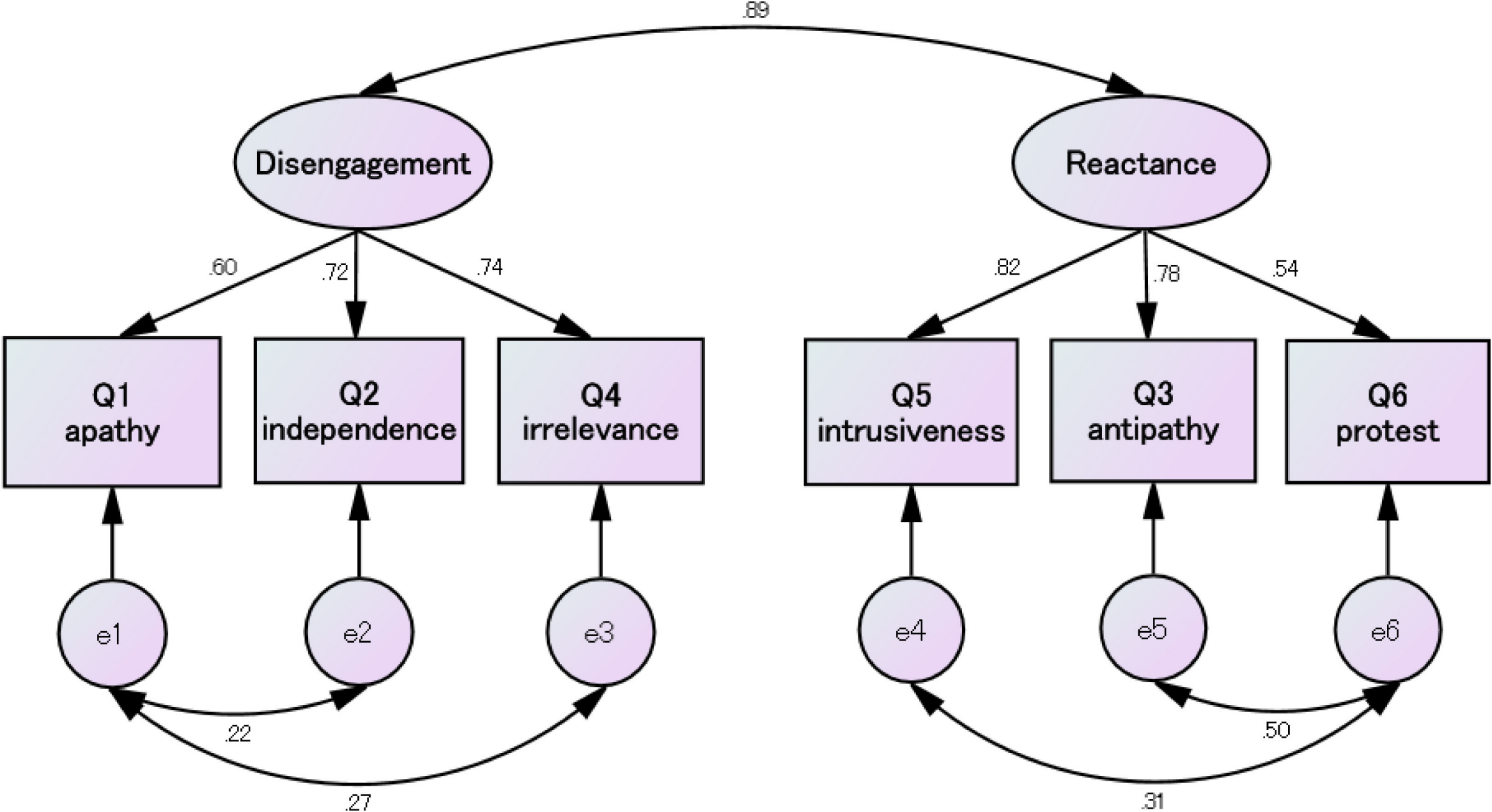
Path diagrams of the two-factor model Rectangles are observed variables (items); ellipses are latent variables (factors); values on the single-headed arrows are standardized factor loadings; and values on the double-headed arrows are correlation coefficients. Model fitness: chi-square 18.691 (df 4) p < 0.001, comparative fit index = 0.998, standardized root mean square residual = 0.011, root mean square error of approximation = 0.041 (90% confidence interval 0.023–0.060)

Figure 1 shows the path diagrams of the confirmatory factor model. The confirmatory factor analysis showed a good fit of the two-factor model with an CFI = 0.998, SRMR = 0.011, and RMSEA = 0.041. Standardized factor loadings ranged between 0.54–0.82. There was a positive correlation between two latent variables (γ = 0.89, p < 0.001).

Table 4 shows the correlation between the resistance score and emotional responses. The resistance scores showed a moderate positive correlation with displeasure and anger and a weak positive correlation with guilty. The other emotions had a non-significant or very weak correlation coefficient.

**Table 4:** Correlation between the resistance score and emotional responses

	**Mean**	**SD**	**γ**	**p**
Displeasure	2.5	1.1	0.55	<0.001
Anger	2.2	1.0	0.53	<0.001
Guilt	2.3	0.9	0.28	<0.001
Fear	2.7	1.0	0.11	<0.001
Happiness	2.2	0.9	0.07	0.001
Pleasure	2.3	0.9	0.04	0.080
Sadness	2.7	1.0	0.02	0.398
Anxiety	2.8	1.1	0.02	0.408
Surprise	3.1	1.0	−0.11	<0.001

Emotional responses were rated on a scale of 1 (not at all) to 5 (extremely).

**Fig. 2:**
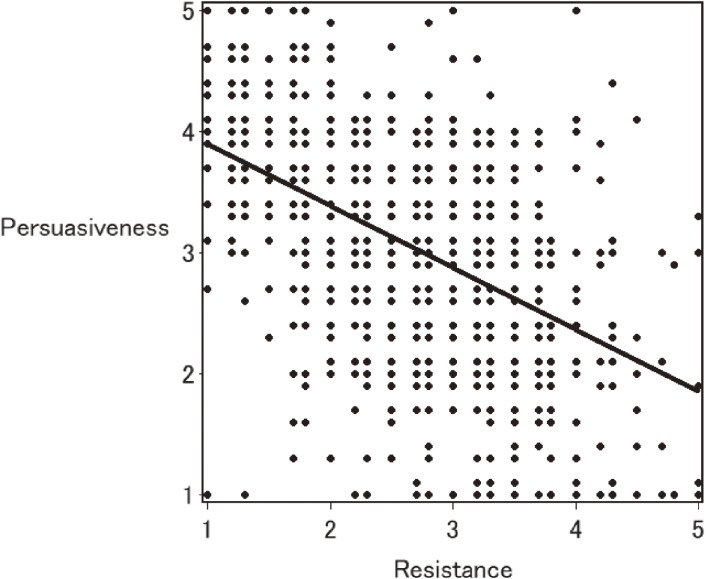
Scatter plot between the reactance score and persuasiveness score Regression line: Reactance score = −0.511 × Persuasiveness score + 4.413

Figure 2 shows the scatter plot between the resistance score and the persuasiveness score. The resistance score was significantly inversely related to the persuasiveness score (γ = −0.50, p < 0.001). Each domain score also showed a negative correlation with the persuasiveness score (the disengagement domain score γ = −0.50, p < 0.001; the reactance domain score γ = −0.40, p < 0.001).

Table 5 shows the association with message attention and ACP intention. Participants who paid greater attention to the message had significantly higher persuasiveness scores and significantly lower resistance scores than those who did not. Participants who expressed a positive ACP intention after seeing the ACP promotion message had significantly higher persuasiveness scores and significantly lower resistance scores than those who did not.

**Table 5:** Association with message attention and ACP intention

	**N**	**Persuasiveness**			**Resistance**		
		**Mean**	**SD**	**p**	**Mean**	**SD**	**p**
Message attention							
Not become aware	343	2.51	0.81	<.0001	3.05	0.78	<.0001
Take no notice	578	2.72	0.65		3.03	0.60	
Throw a glance	1087	3.26	0.60		2.49	0.66	
Stop to look	189	3.68	0.74		2.10	0.79	

ACP intention							
Have no intention	1570	2.85	0.70	<0.001	2.86	0.67	<0.001
Will do	627	3.51	0.68		2.26	0.75	

Persuasiveness and resistance scores were calculated as the average of the scale items scored on a 1-to-5 point Likert scale.

The mean scores were compared using one-way analysis of variance (for message attention) or *t* test (for ACP intention).

The responses to the ACP intention question were dichotomized into “have no intention (I will never do)” and “will do (I have already done/I will do within a month/I will do within six months)”.

Multivariable logistic regression analysis showed that the OR (95% CI) for ACP intention per 1-point increase in the resistance score was 0.47 (0.40–0.56) with adjustment for the persuasiveness score. When each domain score was incorporated into the model instead of the resistance score, the OR (95% CI) was 0.43 (0.37–0.51) for the disengagement domain score and 0.70 (0.61–0.80) for the reactance domain score.

## Discussion

We developed a rating scale measuring active (reactance) and passive (disengagement) resistance to persuasive health messages. The 6-item resistance scale was proven reliable by Cronbach alpha and the two-factor structure corresponding to the disengagement and reactance domains was determined by explanatory factor analysis. The criterion validity was confirmed, as the resistance score was significantly inversely related to the persuasiveness score. The construct validity was confirmed, as those with higher resistance scores were significantly less likely to have a positive ACP intension. These results indicate that the proposed resistance scale is an acceptable, reliable, and valid measure of audience resistance. The scale will be a useful instrument for pretesting persuasive health messages.

Health communication researchers have attempted to explain why message receivers resist being persuaded. The great majority described based on Brehm’s psychological reactance theory, which has been respected in this field over 50 years [10, 11]. However, disengagement was recently identified as another form of audience resistance [12–15]. Disengagement can affect persuasive outcomes independently of reactance. The proposed resistance scale is the first instrument that enables to measure both disengagement and reactance to persuasive health messages simultaneously. Health communication practitioners need to estimate to what extent the intended audience will resist their persuasive messages. The proposed resistance scale can provide them a single score that represents the overall magnitude of resistant reactions to the message. If they want to assess the reactance and disengagement levels separately, the domain scores will be able to meet the request. The use of the scale will help contribute to better understanding and prediction of resistant reactions to persuasive health messages.

Although the phenomenon of reactance has been described with various health messages, the operationalization and measurement of reactance varied widely across studies [10, 11]. Three commonly used measures of state reactance seem to be designed from different aspects: the Dillard and Shen measure operationalized reactance as a combination of anger (as an affective component) and counterargument (as an cognitive component) [18]; the Reactance to Health Warnings Scale consisted of 27 items categorized into anger, self-relevance, common knowledge, exaggeration, government, manipulation, personal attack, derogation, and discounting [19]; and the Salzburger State Reactance Scale consisted of 10 items categorized into experience of reactance, negative attitudes, and aggressive behavioral intentions [20]. Due to the lack of a standard method of measuring reactance, many researchers used a one-off measure whose reliability and validity were not guaranteed.

Unlike reactance, there have been not so many studies on disengagement [12–15]. Disengagement was operationalized as a reverse of attention paid to the message (i.e. inattention), which was measured with several one-off items. It is uncertain whether the inattention items are adequate to fully grasp the essence of disengagement.

In the absence of standard measures of reactance and disengagement, there was no way to examine whether the proposed resistance scale performs better than the standard. The results of this study are not enough to establish the proposed resistance scale as a standard measure of author resistance. However, a comparative study is now ongoing to evaluate many different health messages both on the persuasiveness scale and the resistance scale. We will verify that these two measures have wide applicability to all sorts of health messages, and we will be able to confirm the practicability of the scales for pretesting.

Responses to persuasive messages are likely to differ by cultural and ethnic backgrounds. Unfortunately, research evidence in this area is scarce especially in Japan. Further studies are needed to identify cultural and ethnic differences in responses to persuasive messages. The resistance scale as well as the persuasiveness scale will contribute to the advancement of health communication research.

The results of this study give assurance that the 6-item resistance scale is reliable and valid. On the contrary, this study has the following potential limitations. First, the study participants were recruited from an online research panel. People who cannot access the website through computers or smartphones had no opportunity to participate in the survey. Consequently, the study participants included highly educated people twice as many as in the general population. Although the distribution of HLS-14 scores (i.e. measurements of generic health literacy) in the study participants is quite similar to that obtained from our previous paper-based survey in a Japanese healthcare facility (data not shown) [35], the results of this study may not have reflected responses from people with low educational attainment. Second, the scale items were generated based on literature review. Although the 6-item resistance scale exhibited adequate reliability and validity for measuring audience resistance, it is impossible to deny the possibility that more appropriate set of items would be found by using the thought-listing technique (i.e. a method of collecting cognitive responses from individuals by asking an open-ended question) [36]. Third, the assessment of the scale was conducted only once. We did not compare differences between two or more health messages. Additional assessments of various health messages and in different populations are needed to ensure the generalizability of findings.

## Conclusion

The 6-item resistance scale proposed is the first instrument that enables to measure both active (reactance) and passive (disengagement) resistance to persuasive health messages simultaneously. The scale exhibited adequate reliability and validity for measuring audience resistance when applied to the ACP promotion messages in Japanese people. Health communication researchers and practitioners need to estimate to what extent the intended audience will accept their persuasive messages. The resistance scale can provide them a single score that represents the overall magnitude of resistant reactions to the message. The resistance scale as well as the persuasiveness scale will be useful for pretesting health messages to make them more acceptable to the intended audience, and also they will contribute to the advancement of health communication research.

(The resistance scale developed can be used free of charge for non-commercial projects.)

## Abbreviations

ACP: advance care planning
CFI: comparative fit index
CI: confidence interval
OR: odds ratio
SRMR: standardized root mean square residual
RMSEA: root mean square error of approximation

## Supplementary information

**Additional file 1:** **Appendix:** Resistance scale for Japanese people.

## References

[1] World Health Organization. Advice for the public: Coronavirus disease (COVID-19). https://www.who.int/emergencies/diseases/novel-coronavirus-2019/advice-for-public. Accessed 31 Jan 2022.

[2] Anaki D, Sergay J. Predicting health behavior in response to the coronavirus disease (COVID-19): worldwide survey results from early March 2020. PLoS One. 2021;16:e0244534. 33411827 10.1371/journal.pone.0244534 PMC7790278

[3] Fujii R, Suzuki K, Niimi J. Public perceptions, individual characteristics, and preventive behaviors for COVID-19 in six countries: a cross-sectional study. Environ Health Prev Med. 2021;26:29. 33657995 10.1186/s12199-021-00952-2 PMC7928175

[4] Urbán R, Király O, Demetrovics Z. Who complies with coronavirus disease 2019 precautions and who does not? Curr Opin Psychiatry. 2021;34:363–8. 34001700 10.1097/YCO.0000000000000723 PMC8183252

[5] Sprengholz P, Betsch C, Böhm R. Reactance revisited: consequences of mandatory and scarce vaccination in the case of COVID-19. Appl Psychol Health Well Being. 2021;13:986–95. 34032388 10.1111/aphw.12285 PMC8239828

[6] Lu F, Sun Y. COVID-19 vaccine hesitancy: the effects of combining direct and indirect online opinion cues on psychological reactance to health campaigns. Comput Human Behav. 2022;127:107057. 34707328 10.1016/j.chb.2021.107057 PMC8532517

[7] Ball H, Wozniak TR. Why do some Americans resist COVID-19 prevention behavior? An analysis of issue importance, message fatigue, and reactance regarding COVID-19 messaging. Health Commun. 2022; doi: 10.1080/10410236.2021.1920717. 33941005

[8] Wood S, Schulman K. When vaccine apathy, not hesitancy, drives vaccine disinterest. JAMA. 2021;325:2435–6. 34076690 10.1001/jama.2021.7707

[9] Wood S, Pate MA, Schulman K. Novel strategies to support global promotion of COVID-19 vaccination. BMJ Glob Health. 2021;6:e006066. 10.1136/bmjgh-2021-006066 PMC8521672 34649869

[10] Rosenberg BJ, Siegel JT. A 50-year review of psychological reactance theory: do not read this article. Motiv Sci. 2018;4:281–300.

[11] Reynolds-Tylus T. Psychological reactance and persuasive health communication: a review of the literature. Front Commun. 2019;4:56.

[12] Kim S, So J. How Message fatigue toward health messages lads to ineffective persuasive outcomes: examining the mediating roles of reactance and inattention. J Health Commun. 2018;23:109–16. 29272208 10.1080/10810730.2017.1414900

[13] Martinez Gonzalez A, Reynolds-Tylus T, Quick BL, Skurka C. Message fatigue and resistance to anti-binge drinking messages: examining the mediating roles of inattention and reactance. J Stud Alcohol Drugs. 2021;82:503–10. 34343082

[14] So J. Counterproductive effects of overfamiliar antitobacco messages on smoking cessation intentions via message fatigue and resistance to persuasion. Psychol Addict Behav. 2021; doi: 10.1037/adb0000776. 34914408

[15] Reynolds-Tylus T, Lukacena KM, Truban O. Message fatigue to bystander intervention messages: examining pathways of resistance among college men. Health Commun. 2021;36:1759–67. 32716658 10.1080/10410236.2020.1794551

[16] National Cancer Institute. Making Health Communication Programs Work (Pink Book). https://www.cancer.gov/publications/health-communication. Accessed 31 Jan 2022.

[17] Yzer MC, LoRusso S, Nagler RK. On the conceptual ambiguity surrounding perceived message effectiveness. Health Commun. 2015;30:125–34. 25470437 10.1080/10410236.2014.974131 PMC4677831

[18] Dillard JP, Shen L. On the nature of reactance and its role in persuasive health communication. Commun Monogr. 2005;72:144–68.

[19] Hall MG, Sheeran P, Noar SM, Ribisl KM, Bach LE, Brewer NT. Reactance to health warnings scale: development and validation. Ann Behav Med. 2016;50:736–50. 27333895 10.1007/s12160-016-9799-3 PMC5055422

[20] Sittenthaler S, Traut-Mattausch E, Steindl C, Jonas E. Salzburger State Reactance Scale (SSR Scale). Z Psychol. 2015;223:257–66. 27453806 10.1027/2151-2604/a000227 PMC4957539

[21] Sudore RL, Lum HD, You JJ, Hanson LC, Meier DE, Pantilat SZ, Matlock DD, Rietjens JAC, Korfage IJ, Ritchie CS, Kutner JS, Teno JM, Thomas J, McMahan RD, Heyland DK. Defining advance care planning for adults: a consensus definition from a multidisciplinary delphi panel. J Pain Symptom Manage. 2017;53:821–32.e1. 28062339 10.1016/j.jpainsymman.2016.12.331 PMC5728651

[22] Ministry of Health, Labour, and Welfare. Why don’t you try *Jinseikaigi*? (in Japanese) https://www.mhlw.go.jp/stf/newpage_02783.html. Accessed 31 Jan 2022.

[23] Suka M, Yamauchi T, Yanagisawa H. Perceived effectiveness rating scales applied to insomnia help-seeking messages for middle-aged Japanese people: a validity and reliability study. Environ Health Prev Med. 2017;22:69. 29165165 10.1186/s12199-017-0676-x PMC5664822

[24] Imajo S. The interactive effects of importance of freedom and threat to freedom on psychological reactance: U-shape effect of threat in low-prerequisite-for-reactance condition. Jpn J Exp Soc Psychol. 1995;35:102–10. (in Japanese)

[25] Makino K. Effects of humor on psychological reactance. Res Bull Takamatsu Univ. 2000;34:43–52. (in Japanese)

[26] Kodama M, Kawamori H, Takamoto Y, Fukada H. The effects of humor on persuasion and its mechanism. Hiroshima Psychol Res. 2004;4:63–76. (in Japanese)

[27] Takamoto Y, Yoshimi K, Fukada H. Examination of scales measuring reactance traits. Hiroshima Psychol Res. 2005;5:51–68. (in Japanese)

[28] Imajo S. Do high-pressure communications cause reactance or compliance? Annu Bull Inst Psychol Stud Showa Women’s Univ. 2012;14:1–9. (in Japanese)

[29] Ministry of Internal Affairs and Communications. National Census (in Japanese). https://www.e-stat.go.jp/SG1/estat/GL02100104.do?tocd=00200521. Accessed 31 Jan 2022.

[30] Suka M, Hashimoto J. Use of visuals in print media for public health communication. J Inf Commun. 2020;2:46–53. (in Japanese)

[31] Suka M, Hashimoto J. Acceptability of humorous expressions in public health communication. J Inf Commun. 2021;3:13–21. (in Japanese)

[32] Suka M, Yamauchi T, Yanagisawa H. Responses to persuasive messages encouraging professional help seeking for depression: comparison between individuals with and without psychological distress. Environ Health Prev Med. 2019;24:29. 31068125 10.1186/s12199-019-0786-8 PMC6507167

[33] Terwee CB, Bot SD, de Boer MR, van der Windt DA, Knol DL, Dekker J, Bouter LM, de Vet HC. Quality criteria were proposed for measurement properties of health status questionnaires. J Clin Epidemiol. 2007;60:34–42. 17161752 10.1016/j.jclinepi.2006.03.012

[34] Hu L, Bentler PM. Cutoff criteria for fit indexes in covariance structure analysis: conventional criteria versus new alternatives. Struct Equ Modeling. 1999;6:1–55.

[35] Suka M, Odajima T, Okamoto M, Sumitani M, Igarashi A, Ishikawa H, Kusama M, Yamamoto M, Nakayama T, Sugimori H. Relationship between health literacy, health information access, health behavior, and health status in Japanese people. Patient Educ Couns. 2015;98:660–8. 25739344 10.1016/j.pec.2015.02.013

[36] Cacioppo JT, von Hippel W, Ernst JM. Mapping cognitive structures and processes through verbal content: the thought-listing technique. J Consult Clin Psychol. 1997;65:928–40. 9420354 10.1037//0022-006x.65.6.928

