# Supplementary material for: Development of a rating scale for measuring resistance to persuasive health messages
Source: Environ Health Prev Med. 2022 May 21;27:20. doi: 10.1265/ehpm.22-00059 (PMC9251622; doi:10.1265/ehpm.22-00059)
Supplement: Supplementary file 1 — Additional file 1: Appendix: Resistance scale for Japanese people. [file ehpm-27-020-s001.doc]

Appendix: Resistance scale for Japanese people

このポスターについて、あなたはどのように考えましたか。

What do you think about the message?

|  | 全くそう  でない  strongly disagree | あまりそう  でない  disagree | どちらとも  言えない  unsure | まあ  そうだ  agree | 全く  そうだ  strongly agree |
| --- | --- | --- | --- | --- | --- |
| 1）ポスターに示された事柄について興味がない  I am not interested in the topic. | 1 | 2 | 3 | 4 | 5 |
| 2）このようなことを他人から指図されたくない  I do not want other people to tell me that. | 1 | 2 | 3 | 4 | 5 |
| 3）抵抗感や反感を抱いた  I feel resistance or antipathy. | 1 | 2 | 3 | 4 | 5 |
| 4）自分には関係がないことだ  The message is irrelevant to me. | 1 | 2 | 3 | 4 | 5 |
| 5）押しつけがましさを感じた  The message seems intrusive. | 1 | 2 | 3 | 4 | 5 |
| 6）ポスター制作者に抗議したい  I want to protest against the message sender. | 1 | 2 | 3 | 4 | 5 |
